# Supplementary material for: Therapeutic Vaccine in Chronically HIV-1-Infected Patients: A Randomized, Double-Blind, Placebo-Controlled Phase IIa Trial with HTI-TriMix
Source: Vaccines (Basel). 2019 Dec 6;7(4):209. doi: 10.3390/vaccines7040209 (PMC6963294; doi:10.3390/vaccines7040209)
Supplement: Supplementary file 1 [file vaccines-07-00209-s001.pdf]

vaccines

MDPI

Supplementary Data S1: Inclusion and Exclusion Criteria

Inclusion Criteria

In order to be eligible to participate in this study, a patient must meet all of the following criteria:

1. ≥18 years of age;
2. Voluntarily signed informed consent;
3. Proven HIV-1 infection (with documented antibodies against HIV-1 and a detectable plasma HIV-1 RNA before initiation of therapy);
4. On stable treatment with cART regimen (antiretroviral therapy consisting of at least three registered antiretroviral agents) for at least three years;
5. Nadir CD4+ ≥ 350 cells/μL (up to two occasional determinations ≤350 cells/μL are allowed);
6. Current CD4+ cell count ≥ 450 cells/μL;
7. HIV-RNA below 50 copies/mL in the last six months prior to randomization, during at least two measurements (occasional so-called 'blips' ≤500 copies/mL are permitted);
8. If sexually active, willing to use a reliable method of reducing the risk of transmission to their sexual partners during treatment interruption (including PrEP).

a) For heterosexually active females, using an effective method of contraception with partner (combined oral contraceptive pill; injectable or implanted contraceptive; IUD/IUS; consistent record with condoms; physiological or anatomical sterility (in self or partner) from 14 days prior to the first vaccination until four months after the last vaccination.
b) For heterosexually active males, using an effective method of contraception with their partner from the first day of vaccination until four months after the last vaccination.

Exclusion Criteria

A potential participant who meets any of the following criteria will be excluded from participation in this study:

1. Treatment with non-cART regimen prior to cART regimen;
2. Previous failure to antiretroviral and/or mutations conferring genotypic resistance to antiretroviral therapy;
3. Nonsubtype B HIV infection;
4. Active Hepatitis B virus and/or Hepatitis C virus coinfection;
5. History of a CDC class C event
6. Pregnant female (screened with a positive pregnancy test), lactating or intending to become pregnant during the study;
7. Active history of malignancy ≤30 days (extended period on the clinical assessment of the investigator) prior to screening;
8. Active infection with fever (38 °C or above) ≤10 days of screening and/or first vaccination;
9. Therapy with immunomodulatory agents (e.g. systemic corticosteroids), including cytokines (e.g., IL-2), immunoglobulins and/or cytostatic chemotherapy ≤90 days prior to screening. This does not include seasonal influenza, hepatitis B, and/or other travel related vaccines;
10. Congenital, acquired, or induced coagulation disorders, such as thrombocytopenia (thrombocytes < 150×10^9/L) and/or current use of anticoagulant medication (e.g., coumarins, inhibitors of Xa); Usage of NSAIDs (including acetylsalicylic acid) is allowed; however, it is advised to interrupt therapy 10 days ahead of vaccination;
11. Usage of any investigational drug ≤90 days prior to study entry;
12. An employee of the investigator or study site, with direct involvement in the proposed study or other studies under the direction of that investigator or study site, or is a family member of an employee or the investigator

13. Any other condition, which, in the opinion of the investigator, may interfere with the evaluation of the study objectives

Supplementary Data S2: Statistical Analysis

All enrolled participants were included in both an intention-to-treat and safety analysis. Results are displayed as *N* (%) and median, interquartile range (IQR), unless stated otherwise. The primary safety endpoint is described by number and percentage of grade 3 or above AEs. Because of sparse data, no formal comparison was done for local and systemic AEs. For other clinical or laboratory AEs, the Cochran–Mantel–Haenszel test was performed, stratified by site.

The primary immunogenicity endpoint is described by the total magnitude of HTI specific IFN-γ T cell responses as the sum of SFC/10^6 input PBMC to all positive HTI peptide pools and was calculated as $\log_{10}$ (W6 responses) − $\log_{10}$(baseline responses) and log10 (W18 responses) − $\log_{10}$ (baseline responses). To correct for missing data, we performed multiple imputation of immunogenicity results (for W6, W18) for the primary efficacy endpoint. We randomly imputed 10 datasets, performed statistical analysis on each imputed data set, and obtained final effect estimates by combining results using Rubin's rules. The multiple imputation models contained the following variables: site, vaccination group, baseline ELISpot result, baseline CD4 cell count, nadir CD4 cell count, baseline viral load. The primary efficacy endpoint was compared between groups using a linear mixed effects regression with fixed effect for intervention group and random intercept by site. Analogous analyses were done for IN and OUT peptide pools. All analyses were performed two-sided at 5% significance level, unless otherwise specified. In case an analysis comparing the three intervention groups yielded a significant result ($p < 0.05$), in a second step the three groups were compared pairwise with Bonferroni correction.

Time until viral rebound, evolution of pVL and viral reservoir were analyzed using a linear mixed effects model to compare the evolution over the study period. The models included (nested) random intercepts for subject and site and fixed effects for randomization group and the interaction term between time and randomization group. Effect of intervention group will be tested with type 3 tests of the main group effect and of the interaction term in this model. The models will use linear effects for changes over time.

Sample Size Calculation

Assuming a standard deviation of 0.5 $\log_{10}$ in the change of cumulative frequencies of HIV-specific T cells from baseline, a type 1 error of 5%, a power of 90%, as determined by a two-sided nonparametric test (15% additional subjects), taking into account the weighting for case group ratio of more than 2:1, a sample of 40 in the HTI-TriMix arm, and 15 participants in both TriMix and WFI-arm will allow to detect a difference of at least 0.7 log10 HTI-specific T cell frequencies between any HTI-TriMix arm and the other two arms, allowing for 10% of nonassessable patients.

Interim Analysis

At the time of study design, the moderate increase in T cell responses to peptides spanning the HTI sequence at week 8 of the phase I clinical trial led to extra vigilance. Therefore, we introduced a protocol amendment in order to perform a futility analysis during the phase IIa trial. Recruitment was paused after half of the participants were enrolled, followed by an interim analysis on the primary endpoints of the study, performed by an independent statistician. Immunogenicity results at week 6 of the study were probed to show a 0.7log increase in the HTI-TriMix arm, compared to the WFI-arm. In case standard test statistics for comparing two means was smaller than 2.23, the trial should be stopped for futility; the power of the trial was calculated as 85% in this case.

Supplementary Data S3. Overview of Adverse Events (MedDRA Version 20.0, March 1st, 2017, high level terms)

All patients experienced adverse events (3.1). Patient counts for serious AEs and all drug-related AEs are in 3.2 and 3.3, respectively. None of the patients developed a drug-related serious adverse event. Differences between intervention groups with respect to AE counts (body system totals and common AEs only) were tested using the Cochran–Mantel–Haenszel test, stratified by site.

Supplementary Data 3.1 All patients Experienced Adverse Events

	-- PLACEBO_WFI -- *N* = 8 *n* (%)	-- PLACEBO_TRIMIX -- *N* = 9 *n* (%)	-- VERUM_HIVACAT_TRIMIX -- *N* = 16 *n* (%)	*p*-value
Any AE	8 (100)	9 (100)	16 (100)	
Blood and lymphatic system disorders	1 (12.5)	1 (11.1)	0 (0.0)	0.26
Lymphadenopathy	1 (12.5)	1 (11.1)	0 (0.0)	0.26
Ear and labyrinth disorders	1 (12.5)	0 (0.0)	0 (0.0)	0.37
Vertigo	1 (12.5)	0 (0.0)	0 (0.0)	0.37
Eye disorders	0 (0.0)	0 (0.0)	1 (6.3)	
Vitreous floaters	0 (0.0)	0 (0.0)	1 (6.3)	
Gastrointestinal disorders	6 (75.0)	4 (44.4)	9 (56.3)	0.45
Anal fissure	0 (0.0)	0 (0.0)	1 (6.3)	
Aphthous ulcer	0 (0.0)	0 (0.0)	1 (6.3)	
Colitis	0 (0.0)	0 (0.0)	1 (6.3)	
Diarrhoea	2 (25.0)	4 (44.4)	1 (6.3)	0.064
Dry mouth	0 (0.0)	1 (11.1)	0 (0.0)	0.29
Dyspepsia	1 (12.5)	1 (11.1)	1 (6.3)	0.80
Dysphagia	0 (0.0)	0 (0.0)	1 (6.3)	
Enterocolitis	1 (12.5)	0 (0.0)	1 (6.3)	0.73
Food poisoning	0 (0.0)	1 (11.1)	0 (0.0)	0.22
Nausea	2 (25.0)	0 (0.0)	1 (6.3)	0.23
Oesophageal pain	1 (12.5)	0 (0.0)	2 (12.5)	0.46
Oral dysaesthesia	1 (12.5)	0 (0.0)	0 (0.0)	0.22
Pancreatitis	0 (0.0)	0 (0.0)	1 (6.3)	
Vomiting	1 (12.5)	1 (11.1)	1 (6.3)	0.80
General disorders and administration site conditions	4 (50.0)	5 (55.6)	10 (62.5)	0.80
Asthenia	1 (12.5)	1 (11.1)	1 (6.3)	0.82
Chills	1 (12.5)	1 (11.1)	2 (12.5)	0.98
Cyst	0 (0.0)	0 (0.0)	1 (6.3)	
Fatigue	2 (25.0)	3 (33.3)	2 (12.5)	0.46
Influenza like illness	0 (0.0)	1 (11.1)	1 (6.3)	

Infusion site extravasation	1 (12.5)	1 (11.1)	0 (0.0)	0.41
Injection site pain	2 (25.0)	2 (22.2)	3 (18.8)	0.95
Injection site pruritus	1 (12.5)	0 (0.0)	1 (6.3)	0.49
Injection site reaction	0 (0.0)	1 (11.1)	1 (6.3)	0.52
Malaise	1 (12.5)	1 (11.1)	3 (18.8)	0.79
Pyrexia	1 (12.5)	0 (0.0)	1 (6.3)	0.58
Tenderness	0 (0.0)	1 (11.1)	0 (0.0)	0.082
Immune system disorders	0 (0.0)	0 (0.0)	1 (6.3)	0.51
Hypersensitivity	0 (0.0)	0 (0.0)	1 (6.3)	
Infections and infestations	4 (50.0)	6 (66.7)	9 (56.3)	0.84
Conjunctivitis	0 (0.0)	1 (11.1)	0 (0.0)	0.082
Eyelid infection	0 (0.0)	1 (11.1)	0 (0.0)	0.37
Furuncle	0 (0.0)	0 (0.0)	1 (6.3)	
Gastroenteritis	0 (0.0)	0 (0.0)	2 (12.5)	0.37
Gingivitis	0 (0.0)	1 (11.1)	0 (0.0)	0.22
Hepatitis viral	0 (0.0)	0 (0.0)	1 (6.3)	
Influenza	1 (12.5)	0 (0.0)	0 (0.0)	0.37
Laryngitis	1 (12.5)	0 (0.0)	0 (0.0)	0.22
Lung infection	1 (12.5)	0 (0.0)	0 (0.0)	0.29
Oral herpes	0 (0.0)	0 (0.0)	1 (6.3)	
Respiratory tract infection	0 (0.0)	0 (0.0)	1 (6.3)	
Sexually transmitted disease	0 (0.0)	0 (0.0)	1 (6.3)	
Sinusitis	0 (0.0)	1 (11.1)	0 (0.0)	
Skin infection	1 (12.5)	0 (0.0)	1 (6.3)	0.58
Tonsillitis	0 (0.0)	0 (0.0)	1 (6.3)	
Upper respiratory tract infection	0 (0.0)	1 (11.1)	2 (12.5)	0.62
Urethritis	0 (0.0)	0 (0.0)	1 (6.3)	
Urinary tract infection	0 (0.0)	1 (11.1)	0 (0.0)	0.22
Viral upper respiratory tract infection	1 (12.5)	0 (0.0)	4 (25.0)	0.26
Injury, poisoning and procedural complications	0 (0.0)	1 (11.1)	2 (12.5)	0.56
Accidental exposure to product	0 (0.0)	0 (0.0)	1 (6.3)	
Ligament sprain	0 (0.0)	1 (11.1)	0 (0.0)	0.22
Wrist fracture	0 (0.0)	0 (0.0)	1 (6.3)	
Investigations	1 (12.5)	0 (0.0)	2 (12.5)	0.45
Blood creatine phosphokinase increased	1 (12.5)	0 (0.0)	2 (12.5)	0.45
Metabolism and nutrition disorders	0 (0.0)	0 (0.0)	2 (12.5)	0.37
Gout	0 (0.0)	0 (0.0)	1 (6.3)	
Hypoglycaemia	0 (0.0)	0 (0.0)	1 (6.3)	
Musculoskeletal and connective tissue disorders	3 (37.5)	2 (22.2)	8 (50.0)	0.34

Arthralgia	0 (0.0)	0 (0.0)	1 (6.3)	
Back pain	0 (0.0)	0 (0.0)	1 (6.3)	
Groin pain	0 (0.0)	1 (11.1)	0 (0.0)	0.37
Muscle spasms	1 (12.5)	0 (0.0)	0 (0.0)	0.082
Myalgia	2 (25.0)	1 (11.1)	5 (31.3)	0.52
Neck pain	0 (0.0)	0 (0.0)	1 (6.3)	
Nervous system disorders	5 (62.5)	2 (22.2)	8 (50.0)	0.20
Aphasia	0 (0.0)	0 (0.0)	1 (6.3)	
Disturbance in attention	1 (12.5)	0 (0.0)	0 (0.0)	0.22
Dizziness	1 (12.5)	0 (0.0)	1 (6.3)	0.62
Headache	4 (50.0)	1 (11.1)	6 (37.5)	0.16
Hypoaesthesia	1 (12.5)	0 (0.0)	0 (0.0)	0.22
Muscle contractions involuntary	0 (0.0)	0 (0.0)	1 (6.3)	
Neuralgia	0 (0.0)	0 (0.0)	1 (6.3)	
Somnolence	0 (0.0)	0 (0.0)	1 (6.3)	
Syncope	0 (0.0)	1 (11.1)	0 (0.0)	0.37
Psychiatric disorders	2 (25.0)	2 (22.2)	2 (12.5)	0.70
Anxiety	1 (12.5)	0 (0.0)	0 (0.0)	0.29
Burnout syndrome	0 (0.0)	2 (22.2)	0 (0.0)	0.097
Depression	1 (12.5)	0 (0.0)	1 (6.3)	0.62
Insomnia	1 (12.5)	0 (0.0)	1 (6.3)	0.73
Mental disorder	1 (12.5)	0 (0.0)	0 (0.0)	0.37
Nightmare	0 (0.0)	0 (0.0)	1 (6.3)	
Renal and urinary disorders	1 (12.5)	0 (0.0)	0 (0.0)	0.37
Haematuria	1 (12.5)	0 (0.0)	0 (0.0)	0.37
Reproductive system and breast disorders	1 (12.5)	0 (0.0)	1 (6.3)	0.70
Balanoposthitis	0 (0.0)	0 (0.0)	1 (6.3)	
Erectile dysfunction	1 (12.5)	0 (0.0)	0 (0.0)	0.37
Respiratory, thoracic and mediastinal disorders	2 (25.0)	0 (0.0)	1 (6.3)	0.33
Cough	1 (12.5)	0 (0.0)	0 (0.0)	0.37
Dysphonia	1 (12.5)	0 (0.0)	0 (0.0)	0.37
Productive cough	0 (0.0)	0 (0.0)	1 (6.3)	
Skin and subcutaneous tissue disorders	3 (37.5)	3 (33.3)	4 (25.0)	0.84
Eczema	0 (0.0)	0 (0.0)	1 (6.3)	
Erythema	1 (12.5)	1 (11.1)	0 (0.0)	0.42
Night sweats	1 (12.5)	0 (0.0)	1 (6.3)	0.73
Pruritus	0 (0.0)	1 (11.1)	1 (6.3)	0.81
Rash macular	0 (0.0)	1 (11.1)	0 (0.0)	0.082
Skin swelling	1 (12.5)	0 (0.0)	0 (0.0)	0.22

Skin ulcer	0 (0.0)	0 (0.0)	1 (6.3)	
Vascular disorders	0 (0.0)	1 (11.1)	0 (0.0)	0.37
Hypotension	0 (0.0)	1 (11.1)	0 (0.0)	0.37

Supplementary data 3.2 Patient counts for serious AEs

	-- PLACEBO_WFI -- *N* = 8 *n* (%)	-- PLACEBO_TRIMIX -- *N* = 9 *n* (%)	-- VERUM_HIVACAT_TRIMIX -- *N* = 16 *n* (%)	*p*-value
Any AE	1 (12.5)	0 (0.0)	2 (12.5)	0.54
Gastrointestinal disorders	0 (0.0)	0 (0.0)	2 (12.5)	0.31
Colitis	0 (0.0)	0 (0.0)	1 (6.3)	
Pancreatitis	0 (0.0)	0 (0.0)	1 (6.3)	
Psychiatric disorders	1 (12.5)	0 (0.0)	0 (0.0)	0.29
Depression	1 (12.5)	0 (0.0)	0 (0.0)	0.29

Supplementary data 3.3 All drug-related AEs

	-- PLACEBO_WFI -- *N* = 8 *n* (%)	-- PLACEBO_TRIMIX -- *N* = 9 *n* (%)	-- VERUM_HIVACAT_TRIMIX -- *N* = 16 *n* (%)	*p*-value
Any AE	6 (75.0)	8 (88.9)	10 (62.5)	0.37
Blood and lymphatic system disorders	0 (0.0)	1 (11.1)	0 (0.0)	
Lymphadenopathy	0 (0.0)	1 (11.1)	0 (0.0)	
Gastrointestinal disorders	3 (37.5)	0 (0.0)	1 (6.3)	0.050
Diarrhoea	1 (12.5)	0 (0.0)	0 (0.0)	0.22
Dysphagia	0 (0.0)	0 (0.0)	1 (6.3)	
Nausea	2 (25.0)	0 (0.0)	0 (0.0)	0.065
General disorders and administration site conditions	4 (50.0)	5 (55.6)	7 (43.8)	0.90
Asthenia	1 (12.5)	1 (11.1)	1 (6.3)	0.82
Chills	1 (12.5)	1 (11.1)	1 (6.3)	0.91
Fatigue	2 (25.0)	3 (33.3)	1 (6.3)	0.22
Influenza like illness	0 (0.0)	0 (0.0)	1 (6.3)	
Infusion site extravasation	1 (12.5)	1 (11.1)	0 (0.0)	0.41
Injection site pain	2 (25.0)	1 (11.1)	3 (18.8)	0.68

Injection site pruritus	1 (12.5)	0 (0.0)	1 (6.3)	0.49
Injection site reaction	0 (0.0)	1 (11.1)	1 (6.3)	0.52
Malaise	1 (12.5)	1 (11.1)	2 (12.5)	0.86
Pyrexia	1 (12.5)	0 (0.0)	1 (6.3)	0.58
Infections and infestations	1 (12.5)	0 (0.0)	3 (18.8)	0.39
Hepatitis viral	0 (0.0)	0 (0.0)	1 (6.3)	
Laryngitis	1 (12.5)	0 (0.0)	0 (0.0)	0.22
Oral herpes	0 (0.0)	0 (0.0)	1 (6.3)	
Viral upper respiratory tract infection	0 (0.0)	0 (0.0)	1 (6.3)	
Investigations	0 (0.0)	0 (0.0)	1 (6.3)	
Blood creatine phosphokinase increased	0 (0.0)	0 (0.0)	1 (6.3)	
Musculoskeletal and connective tissue disorders	3 (37.5)	2 (22.2)	3 (18.8)	0.59
Groin pain	0 (0.0)	1 (11.1)	0 (0.0)	0.37
Muscle spasms	1 (12.5)	0 (0.0)	0 (0.0)	0.082
Myalgia	2 (25.0)	1 (11.1)	2 (12.5)	0.77
Neck pain	0 (0.0)	0 (0.0)	1 (6.3)	
Nervous system disorders	3 (37.5)	1 (11.1)	4 (25.0)	0.43
Disturbance in attention	1 (12.5)	0 (0.0)	0 (0.0)	0.22
Dizziness	1 (12.5)	0 (0.0)	0 (0.0)	0.22
Headache	2 (25.0)	1 (11.1)	4 (25.0)	0.60
Hypoaesthesia	1 (12.5)	0 (0.0)	0 (0.0)	0.22
Skin and subcutaneous tissue disorders	2 (25.0)	2 (22.2)	1 (6.3)	0.37
Erythema	1 (12.5)	1 (11.1)	0 (0.0)	0.42
Pruritus	0 (0.0)	1 (11.1)	1 (6.3)	0.81
Skin swelling	1 (12.5)	0 (0.0)	0 (0.0)	0.22
Vascular disorders	0 (0.0)	1 (11.1)	0 (0.0)	0.37
Hypotension	0 (0.0)	1 (11.1)	0 (0.0)	0.37

Supplementary Data S4. Plasma Viral Load and Ultrasensitive Plasma Viral Load

Panel A: Plasma viral loads (pVL) in Log_{10} copies/mL relative to the stop of ART ("0")
Panel B: pVL in Log_{10} copies/mL relative to the restart of ART (end of ATI)
Panel C: Ultrasensitive pVL in Log_{10} copies/mL to quantify changes in pVL induced by the vaccinations below a threshold of <50 copies/mL.

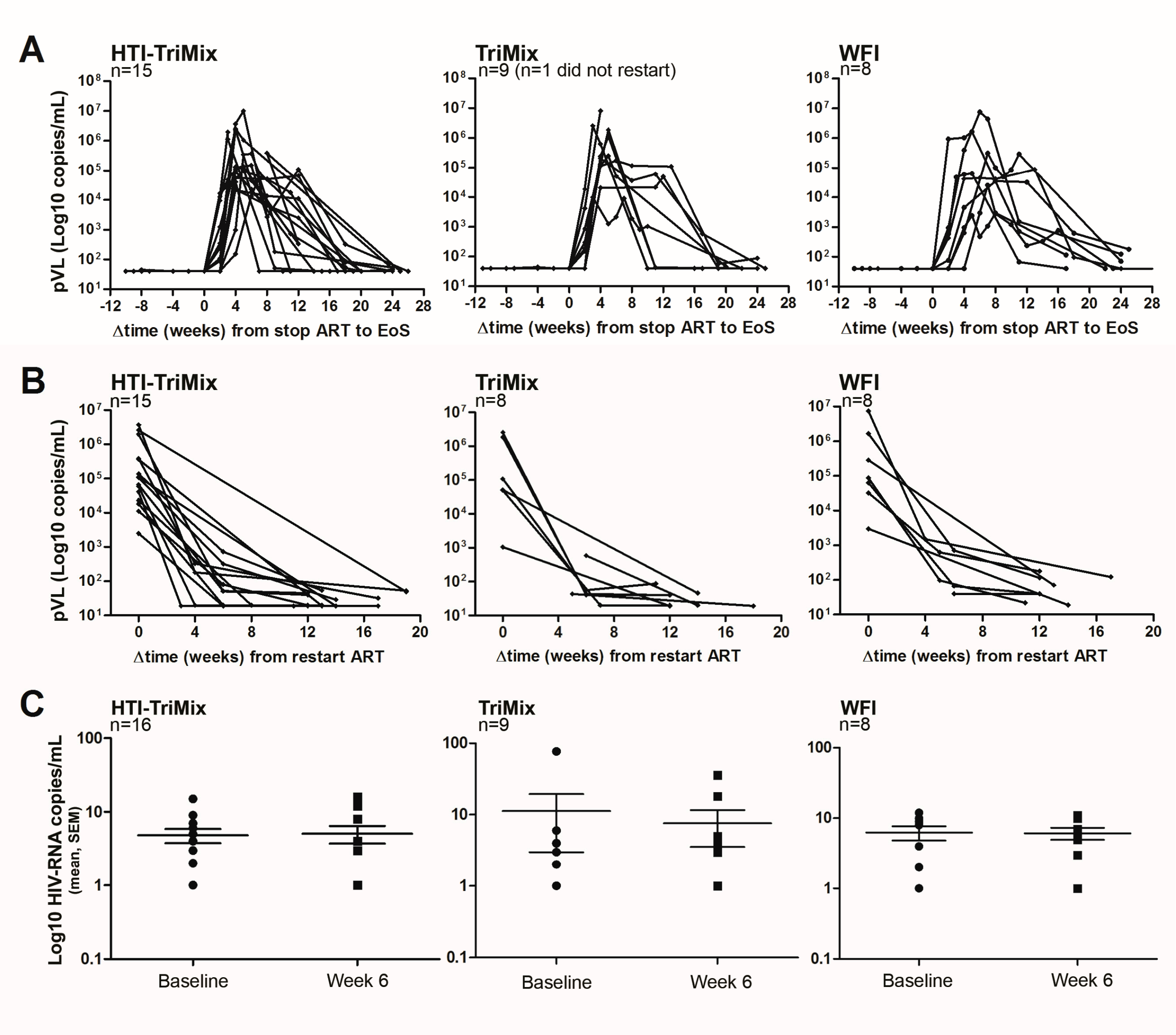

Supplementary Data S5. Viral Inhibition Assay

Methods:

CD8 T cell-mediated viral suppression was measured on PBMCs derived from screening, baseline and week 4 with a viral inhibition assay (VIA) as described previously [1] with minor modifications. For the preparation of CD4+ T target cells, PBMCs were stimulated during seven days at 37 °C 7% CO_2 in RPMI 2.5% human serum (HS, A&E Scientific, Belgium), IL-2 (500 IU/mL, Gentaur, Kampenhout, Belgium) and antihuman CD3/8 bispecific monoclonal antibody (1 µg/mL, NIH AIDS Reagent Program). For the preparation of stimulated CD8+ T effector cells, PBMCs were incubated for seven days at 37 °C 7% CO_2 in RPMI 2.5% HS and 28.6 µg/mL (200 ng/mL per peptide) of the HTI peptide pool. For the preparation of nonstimulated CD8+ T effector cells, an extra aliquot of PBMCs was thawed one day before the start of CD4+/CD8+ T cell coculture and rested overnight at 37 °C 7% CO_2 in RPMI 2.5% HS.

On day 0 of the VIA, CD4+ T target cells were further enriched by negative selection using magnetic beads (Miltenyi Biotech, San Diego, CA, USA) from the anti-CD3/8 mAb stimulated PBMCs. Stimulated as well as nonstimulated CD8+ T effector cells were enriched from the HTI peptide pool stimulated PBMCs and overnight rested PBMCs respectively, by negative selection. Cell purities of enriched CD4+ T and CD8+ T cells of >90% were confirmed by staining for CD3 (FITC, Clone OKT3), CD4 (PE, clone SK3), CD8a (APC-eFluor, clone SK1) and reading on a BD FACSVerse (Beckton Dickinson, New Jersey, USA).

Enriched activated CD4+ T cells were infected with HIV-1 HTLVIIIB at a multiplicity of infection of 0.001 for three hours at 37 °C 7% CO_2. Infected target cells were washed three times and resuspended at 10^6 cells/mL in RPMI 2.5% HS 500 IU/mL IL-2 and cultured in triplicate in flat bottom 96-well plates (VWR, Leuven, Belgium) at 10^5 cells/well, alone (positive control) or in coculture with stimulated or rested enriched CD8+ effector T cells at an effector-to-target ratio of 0.1:1 and 2:1. Culture medium was refreshed at days 2, 6, and 9. The level of HIV-1 p24 antigen in the supernatant was determined at day 13 by in-house p24 ELISA. Log inhibition values were calculated on day 13 as $\log_{10}$(p24 without CD8) − $\log_{10}$(p24 with CD8).

Statistical Analysis:

The CD8+ T cell HIV suppressive capacity (viral inhibition essay) is represented as ($\log_{10}$(virus|CD4) − $\log_{10}$(virus|CD4/CD8)). The evolution of CD8+ T cell HIV suppressive capacity from baseline until week 4 will be compared between the three intervention groups using mixed effects linear regression.

1 | Pannus P, Adams P, Willems E, Heyndrickx L, Florence E, Rutsaert S, et al. In-vitro viral suppressive capacity correlates with immune checkpoint marker expression on peripheral CD8+ T cells in treated HIV positive patients. AIDS. 2019 Mar 1;33(3):387-398.

Results:

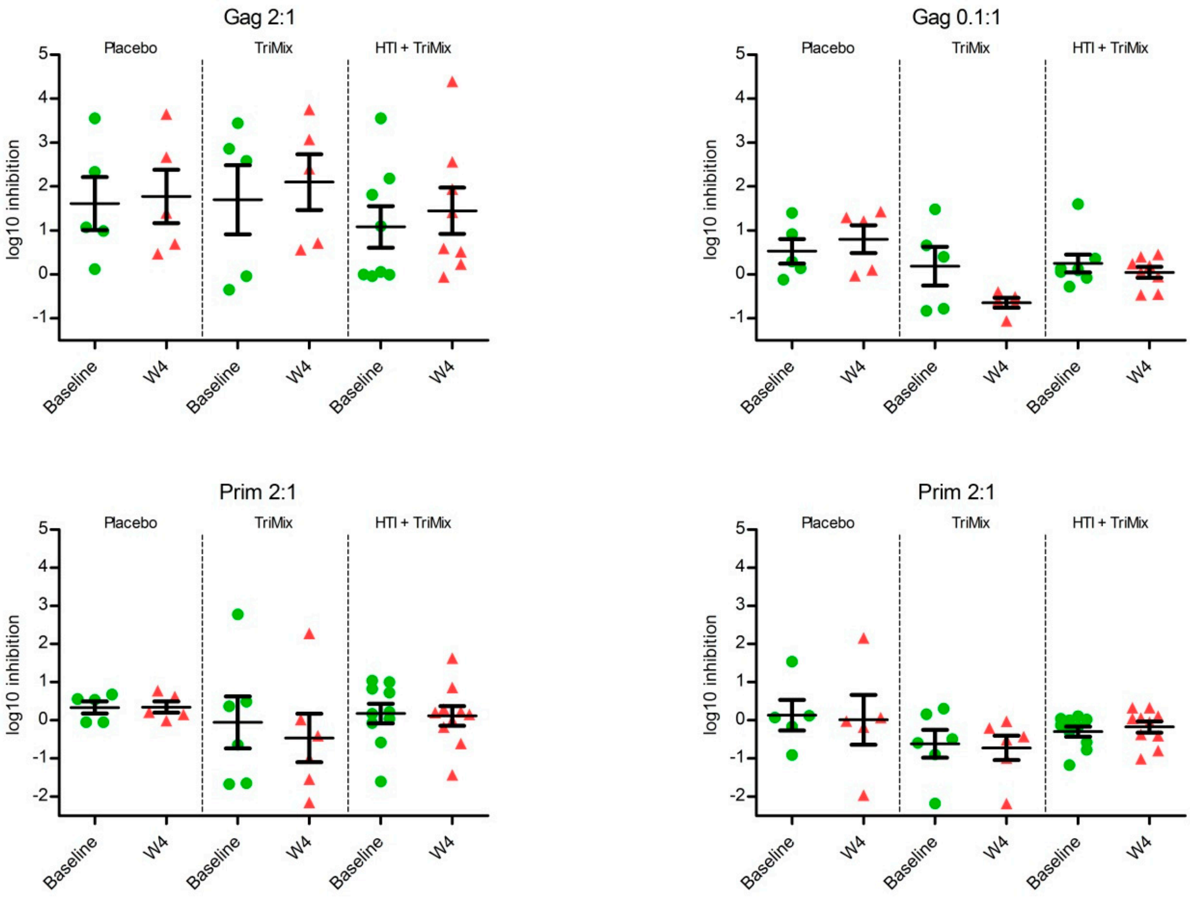

Placebo = water for injection (WFI). Gag = Gag peptide stimulation.

The change in CD8 T cell HIV suppressive capacity ($\log_{10}$(virus|CD4) − $\log_{10}$(virus|CD4/CD8)) from baseline until week 4 is shown above. For the restimulated CD8 T cells with a CD8:CD4 ratio of 0.1:1, the evolution over time was significantly lower both in the HTI-TriMix and the TriMix arm compared to the WFI arm.

Condition	Intervention arm	Mean change from baseline	Mean difference (95% CI)
Ex vivo (2:1)			(*p*-value = 0.21)
	PLACEBO_WFI	0.07	Reference category
	PLACEBO_TRIMIX	-0.54	-0.60 (-1.36, 0.16)
	VERUM_HIVACAT-TRIMIX	-0.04	-0.10 (-0.78, 0.57)
Ex vivo (0.1:1)			(*p*-value = 0.55)
	PLACEBO_WFI	-0.05	Reference category
	PLACEBO_TRIMIX	-0.15	-0.09 (-0.70, 0.51)
	VERUM_HIVACAT-TRIMIX	0.12	0.17 (-0.37, 0.72)
Stimulated (2:1)			(*p*-value = 0.89)
	PLACEBO_WFI	0.18	Reference category
	PLACEBO_TRIMIX	0.40	0.22 (-0.73, 1.16)
	VERUM_HIVACAT-TRIMIX	0.33	0.15 (-0.69, 0.99)
Stimulated (0.1:1)			(*p*-value = 0.002)
	PLACEBO_WFI	0.41	Reference category
	PLACEBO_TRIMIX	-0.91	-1.32 (-2.00,-0.65)
	VERUM_HIVACAT-TRIMIX	-0.24	-0.65 (-1.26,-0.05)
